# Endoscopic and Histologic Predictors of Outcomes in Pediatric Ulcerative Colitis—*Caveat Emptor*

**DOI:** 10.3389/fped.2021.678132

**Published:** 2021-06-23

**Authors:** Lorraine Stallard, Séamus Hussey

**Affiliations:** ^1^National Centre for Paediatric Gastroenterology, Children's Health Ireland at Crumlin, Dublin, Ireland; ^2^Department of Paediatrics, Royal College of Surgeons of Ireland and University College Dublin, Dublin, Ireland; ^3^DOCHAS Study, National Children's Research Centre, Dublin, Ireland

**Keywords:** ulcerative colitis, pediatrics, endoscopy, histology, mucosal healing

## Abstract

The impact of endoscopic and histological mucosal healing on outcomes in adult settings is impressive. Despite many clinical parallels, pediatric ulcerative colitis (UC) is set apart from adult disease in several respects. Many frequently used indices are not fully validated, especially in pediatric settings, and consensus on precise definitions in clinical settings are lacking. Endoscopic mucosal healing is an acceptable long-term treatment goal in pediatrics, but not histologic normalization. Early prediction of disease course in UC may allow treatment stratification of patients according to risks of relapse, acute severe colitis, and colectomy. Putative endoscopic and histologic predictors of poor clinical outcomes in adults have not held true in pediatric settings, including baseline endoscopic extent, endoscopic severity, and specific histologic characteristics which are less prevalent in pediatrics at diagnosis. In this mini-review we appraise predictive endoscopic and histologic factors in pediatric UC with reference to relapse, severe colitis, and colectomy risks. We recommend that clinicians routinely use endoscopic and histologic sores to improve the quality of clinical and research practice. The review summarizes differences between adult and pediatric prediction data, advises special consideration of those with primary sclerosing cholangitis, and suggests areas for future study in this field.

## Core Tip

Predicting risks of relapse, severe colitis, and colectomy in adults using endoscopic and histologic data has shown early promise. The tools used have not undergone pediatric validation. Unlike adult patients, endoscopic severity poorly predicts short- and medium-term risks of relapse, medication use, and colectomy in pediatrics. Histologic features associated with poorer short- to medium-term outcomes in pediatric UC include low rectal eosinophil counts and surface villiform changes at diagnosis. Stool biomarkers may identify children with endoscopic healing, a long-term treatment goal.

## Introduction

Ulcerative colitis (UC) is a chronic inflammatory intestinal disorder, with a relapsing and remitting course. Approximately 25% of all IBD presents by late adolescence, and a recent sustained rise in incidence over time has been observed, particularly in Western countries (1, 2). While many patients successfully respond to first line treatments, others will progress through medically refractory disease to colectomy. Pediatric UC shares much in common with adult disease, but distinctive pediatric features of phenotype, histopathology, and clinical course are well described. As many as 70–80% of children are diagnosed with either extensive colitis or pancolitis compared with only 20–40% of adults (3). Pediatric UC has more histologically active inflammation but less chronicity features than adult disease at diagnosis (4). Pediatric disease is more often associated with severe exacerbations, is proportionally more aggressive, and requires more intensive pharmacological and surgical treatments than adult phenotypes (5–7).

Many clinicians now use “mucosal healing” as their treatment target, appreciating that patients in clinical remission frequently have residual endoscopic and histologic activity (8–10). Although many use a Mayo Clinic Endoscopic Score (MES) of ≤1 to define mucosal healing in UC clinical trials, formal consensus has not been reached on its optimal definition, and histologically active disease is associated with greater risk of subsequent relapse (11, 12). Recent studies estimate that between 14 and 40% of patients with an endoscopically normal mucosa have ongoing histologic activity (10, 11, 13, 14). Integration of endoscopic and histological grading, using advanced endoscopic imaging modalities, may help bridge the void between these two domains (15–17).

Treatment decisions, once mainly influenced by disease activity, increasingly incorporate adverse outcome mitigation in the process. Early predictors for those most likely to have worst outcomes would help guide treatment and tailor therapy to the individual patient (18). The PIBD Ahead Programme recently published an extensive appraisal and statement of current outcome predictors in UC (19). Among the outcomes most concerning to clinicians in a survey across 33 countries were risk of relapse, developing acute severe colitis and colectomy. Many studies have been hindered by their retrospective nature, combined with a lack of standardized treatment protocols (20, 21). Nonetheless, characteristics including initial clinical disease severity, disease extent, hemoglobin, albumin, inflammatory markers, and genetic polymorphisms have shown limited, if any, reproducibility in predicting clinical outcomes of relevance (22–26).

Predicting those at risk of poorer outcomes using endoscopic and histologic markers would allow bespoke treatments to meet patients' needs, but studies to date show conflicting and variable results (27, 28). In this mini-review we appraise the putative endoscopic and histologic predictors of outcomes in pediatric UC, focusing on the most important clinical outcomes of relapse, severe UC, and risk of surgery. We highlight the limitations of current data, differences between pediatric and adult outcome data (see Table 1), suggest a cautious approach in utilizing such data in clinical practice and identify areas of opportunities to address current knowledge gaps for future clinical pathways.

**Table 1:** Outcome associations of endoscopic and histologic measures.

**Authors**	**Year of publication**	**Study type**	**Key variables**	**Index used**	**Key outcome/Finding**
Turner et al. (21)	2013	Prospective RCT	a) Baseline MES b) Week 8, MES-0	MES	a)⦸ 1-year SSFR b) ↑ 1-year SSFR
Lascurain et al. (29)	2016	Retrospective cohort ± PSC	Endoscopic severity	MES	⦸-colectomy
Schechter et al. (30)	2015	Retrospective inception cohort	↑ baseline MES	MES; study-specific histology score; PUCAI	↑ ASC ⦸ Sustained remission ⦸ Colectomy
Boyle et al. (31)	2017	Prospective observational cohort	↓ Eosinophil density; Villiform surface	MES; study-specific histology score; PUCAI	↑ Clinical severity
Hyams et al. (20)	2017	Prospective observational cohort	a) Low Eosinophil density; b) Low MES	MES; study-specific histology score; PUCAI	a) ↓ Short-term outcomes b) ↑ Week 4 remission
Hyams et al. (32)	2019	Prospective observational cohort	a) ↓ Eosinophil density; b) Mild endoscopic disease	MES; study-specific histology score; PUCAI	a) ↑ Risk anti-TNFα escalation b) ↑ Week 52 SSFR
Ricciuto et al. (33)	2020	Retrospective cohort	Baseline endoscopic and histologic severity	MES, Geboes	In PSC, PUCAI does not correlate with endoscopic severity
Gupta et al. (34) (adults)	2020	Systematic review and meta-analysis	a) Residual histologic activity b) Endoscopic + histologic remission	Multiple indices	a) ↑ Relapse rates b) ↓ Relapse rates
Yoon et al. (35) (adults)	2020	Systematic review and meta-analysis	a) Endoscopic-only remission b) Histologic + endoscopic remission	Multiple indices	a) ↓ Relapse risk b) ↓↓ Relapse risk
Cushing et al. (36) (adults)	2020	Prospective cohort	Histologic normalization (Geboes 0) Endoscopic remission	Geboes Score, Nancy Index, Robart's Index	↓ Relapse rates
D'Amico et al. (37) (adults)	2021	Observational retrospective cohort	Residual histological disease activity	Nancy Index	↑ Surgery risk ↑ Hospitalization

*MES, Mayo Clinic Endoscopic Subscore; PUCAI, Pediatric Ulcerative Colitis Activity Index; RCT, Randomized controlled trial; PSC, primary sclerosing cholangitis; ASC, Acute Severe Colitis; SSFR, Sustained steroid-free remission. ⦸ No association; ↑ increased; ↓ reduced*.

## Endoscopy

Endoscopy is fundamental to the correct assignment of disease phenotype, characterization of mucosal endoscopic severity, obtaining biopsy samples, and monitoring response to treatment. Clinician subjectivity results in broad interpretation of endoscopic findings without standardized approaches. Even widely used endoscopic indices are prone to substantial inter- and intra-rater variability, and all lack a reference standard. Disease phenotype relies on the “geographic” extent of macroscopic disease, rather than severity of appearance (38). Intuition rather than data leads clinicians to infer that endoscopic severity portends worse clinical outcomes.

### Endoscopic Scoring Indices

At least 19 different endoscopic scoring systems have been developed, primarily to define endoscopic disease activity. Two scoring systems stand above the others in terms of extent of formal validation in adult settings (see Table 2) (39). Remarkably, all systems lack complete validation, in adult or pediatric settings. The Mayo Clinic endoscopic subscore (MES) has had widespread use in clinical and trial settings since it was first introduced (41). Its simplicity is a major attraction, with just four different categories. The Ulcerative Colitis Endoscopic Index of Severity (UCEIS) score grades vascular pattern, bleeding, erosions, and ulceration (42). Studies with UCEIS have used the recto-sigmoid segment, the worst affected colonic segment or a composite score for all segments to derive the final score. Despite having no formal assessment of interval grades of mild, moderate, and severe disease, UCEIS has been used in responsiveness settings, and we recommend its use in routine clinical care and research initiatives.

**Table 2:** Strengths and limitations of endoscopic and histologic scores.

**Index**		**Original validation (39)**	**Pediatric validation**	**Predicts relapse**	**Predicts acute severe colitis**	**Predicts colectomy**	**Predicts medication escalation**
**Endoscopy**							
Mayo Clinic Endoscopic Subscore (MES)	0 = normal mucosal appearance 1 = mild friability, reduced vascular pattern, erythema 2 = marked friability, absent vascular pattern, marked erythema, visible erosions 3 = Visible ulceration, spontaneous mucosal bleeding *Remission: MES 0* *Response: Δ MES ≥1*	Reliability + Construct + Criterion + Responsiveness + Feasibility –	Partial	– +^a^	+ +^a^	– +^a^	+/-* (* week 52 steroid free remission) +^a^
Ulcerative Colitis Endoscopic Index of Severity (UCEIS)	**Vascular pattern** 0 = normal appearing vasculature 1 = Patchy loss of vasculature 2 = Complete loss of visible vasculature **Erosions and Ulceration** 0 = normal mucosa, neither erosions nor ulcers 1 = tiny (≤ 5mm) white/yellow mucosal erosions 2 = larger (>5mm) superficial mucosal defects, fibrin-covered ulcerations 3 = Deep mucosal defects with slightly raised edges **Bleeding** 0 = none present 1 = mucosal spots or streaks of blood in advance of the scope; can be washed easily 2 = Mild luminal liquid blood 3 = Moderate or large volume of frank blood in lumen in advance of the scope, or, visible blood oozing from mucosa after washing, or spontaneous blood oozing from mucosa *Remission: UCEIS 0* *Response: ΔUCEIS ≥2*	Reliability + Construct + Criterion + Responsiveness + Feasibility –	Partial	N/A +^a^	N/A +^a^	N/A +^a^	N/A +^a^
**Histology**							
Nancy index	Grade 0 = mild or no chronic inflammatory infiltrate Grade 1 = No acute inflammatory infiltrates, moderate-marked chronic inflammatory infiltrates only Grade 2 = Mild acute inflammatory infiltrate (mildly active disease) Grade3 = No ulceration; moderate-severe acute inflammatory cell infiltrate (moderately active disease) Grade 4 = Ulceration (severely active disease) *Remission: <2* *Response: Δ ≥1 reduction*	Reliability + Content + Responsiveness + Feasibility –	N/A	+^a^	+^a^	+^a^	+^a^
Robarts index	**Chronic inflammatory infiltrate** 0 = No increase 1 = Mild but unequivocal increase 2 = Moderate increase 3 = Marked increase **Lamina propria neutrophils** 0 = None 1 = Mild but unequivocal increase 2 = Moderate increase 3 = Marked increase **Neutrophils in epithelium** 0 = None 1 = <5% crypts involved 2 = <50% crypts involved 3 = >50% crypts involved **Erosion or ulceration** 0 = None 1 = Recovering epithelium with adjacent inflammation 1 = Probable erosion—focal 2 = Unequivocal erosion 3 = Ulcer or granulation tissue *Range 0 (no activity) to 33(severe activity*); *Remission ≤ 3* *Response: Δ ≥7reduction*	Reliability + Content + Responsiveness + Feasibility –	N/A	+/−^a^	?^a^	?^a^	+/−^a^
Geboes score (*continuous scale*)	**Architectural changes** 0 = No abnormality 1 = Mild abnormality 2 = Mild or moderate diffuse or multifocal abnormalities 3 = Severe diffuse or multifocal abnormalities **Chronic inflammatory infiltrate** 3 = No increase 4 = Mild but unequivocal increase 5 = Moderate increase 6 = Marked increase **Lamina propria neutrophils and eosinophils** *Eosinophils* 6 = No increase 7 = Mild but unequivocal increase 8 = Moderate increase 9 = Marked increase *Neutrophils* 9 = No increase 10 = Mild but unequivocal increase 11 = Moderate increase 12 = Marked increase **Neutrophils in epithelium** 12 = None 13 = <5% Crypts involved 14 = 5–50% Crypts involved 15 = >50% Crypts involved **Crypt destruction** 15 = None 16 = Probable—local excess of neutrophils in part of crypt 17 = Probable—marked attenuation 18 = Unequivocal crypt destruction **Erosion or ulceration** 18 = None present 19 = Recovering epithelium + adjacent inflammation 20 = Probable erosion; focally stripped 21 = Unequivocal erosion 22 = Ulcer or granulation tissue *Remission: score ≤ 7* *Improvement: score <14*	Reliability + Content, construct + Responsiveness – Feasibility –	partial	N/A +^a^	N/A +/-^a^	+/-^*^ (*surface villiform changes associated with medical escalation or colectomy) +^a^	+/-^*^ (* <32 eosinophils per rectal high powered field associated with anti-TNFα escalation) +^a^

*N/A, not available, not assessed in pediatrics; +, affirmative; –, negative, not tested; +/-, heterogenous findings;*

a *adult data only; Δ, change or “delta”; ?, unknown*.

### Baseline Endoscopy Outcomes

In children, unlike adults, endoscopic severity at diagnosis is not a reliable indicator of post-induction outcomes or future colectomy (43, 44). A multicentre retrospective pediatric inception cohort study of 115 children with new onset UC found that baseline endoscopic severity, disease extent, and PUCAI, were not predictive of sustained steroid free remission (SSFR) or colectomy (30). A baseline MES of 3/3 was found in 36 children (32%), who did not differ from those with Mayo scores of 1–2 regarding the primary outcome of SSFR (*p* = 0.204). The PROTECT (Predicting Response to Standardized Pediatric Colitis Therapy) study (15), a large pediatric multicentre inception cohort, found that a total Mayo clinical and endoscopic score <10 at diagnosis (OR 1.8, 95% CI 1.1–3.0) was associated with a greater likelihood of week flour clinical remission (20). Total Mayo score ≥11 at baseline was a predictor of treatment escalation by week 12 in those initially treated with intravenous corticosteroids (2.6, 0.9–7.2; *p* = 0.068). The subsequent 1-year outcome study showed some evidence that week 52 SSFR was more likely in those with lower MES scores (32). A high MES score in children was associated with development of future acute severe colitis in the Schechter study, but not medication escalation (30).

The UCEIS also predicts risk of relapse, acute severe colitis, medication escalation and colectomy (45, 46). Comparable pediatric UCEIS data has yet to be gathered. Ikeya et al., in a prospective study of 41 adult patients with UC, found that the UCEIS performed better than the MES in predicting relapse free survival (47). Post-induction UCEIS scores were also associated with lower odds of colectomy or relapse. It remains speculative whether the MES or UCEIS would better address the current limitations of risk identification in pediatrics. The higher prevalence of relative rectal sparing in pediatrics may account for some discrepancy from adult data, but structured and centralized reading of endoscopic appearances in future pediatric studies is essential to control for otherwise avoidable variability and weaknesses in endoscopic scoring data.

### Outcomes From Interval Endoscopies

Studies of endoscopic disease extension using the Paris classification are limited and have variable results (38, 48, 49). The EPIMAD cohort found colonic extension from E1 or E2 to E2 or E3 [*HR* = 13.3 (1.7–101.7)] as a risk for colectomy (5). Assa et al. reported that severe disease at diagnosis but not disease extent was associated with risk for colectomy [*HR* = 3.5, *p* = 0.002], hospitalization [*HR* = 3.3, *p* < 0.001], flare [*HR* = 2.4, *p* < 0.001], and biologic therapy [*HR* = 2.6, *p* = 0.001] (7). However, for their study analysis UC was grouped with IBD-unclassified and phenotype extent classes were merged (7). The predictive properties of endoscopic scores may as much relate to the timing of the endoscopic assessment, e.g., post-induction or week 52, as to the score itself. Better clinical outcomes are likely in patients achieving remission post-induction. The T-72 trial of infliximab in ulcerative colitis showed the PUCAI score was reasonably concordant with endoscopic severity using the MES, prompting the conclusion that endoscopic scoring added little to clinical assessment following induction (21). That said, mucosal healing at follow-up sigmoidoscopy was predictive of SSFR at 1 year in that trial. In a prospective study of 82 adult patients, a UCEIS <1 after treatment with infliximab was associated with better long-term outcomes (50). Post-therapeutic UCEIS scores, but not pre-treatment scores, were predictive of short-term outcomes and likely reflected mucosal healing. Interval MES scores in adult settings were also more helpful than baseline scores at identifying patients more likely of adverse outcomes, including relapse, acute severe colitis, hospitalization, and colectomy (44, 51). Timed endoscopic assessments after induction are likely to provide the earliest predictive time-point for patients with UC. Future studies of endoscopic score need to address the questions of optimal timing and best scoring system before clinical practice changes.

### Endoscopic Healing

Mucosal healing is an ideal long-term clinical and therapeutic outcome. In practice, it often remains elusive, and lacks an acceptable and validated definition. Endoscopic healing was associated with favorable 1-year outcomes in children following induction with infliximab (21). Multiple studies in adult patients have found better clinical outcomes regarding relapse, hospitalization, and colectomy among patients with endoscopic healing, using either the MES or UCEIS (43, 44, 47). Repeated endoscopic evaluation is more feasible in adults than children, but fecal biomarkers, such as calprotectin, now guide better patient selection for a timely endoscopic re-evaluation. Validation of thresholds for both endoscopic sores and fecal biomarkers remains an outstanding genuine weakness in this academic field and should be prioritized in future studies.

## Histology

### Histologic Indices

Traditionally, the preserve of histology lay in confirming the hallmarks of ulcerative colitis, and gauging severity. Renewed interest in the prognostic potential of histopathologic features has highlighted deficits in standardized definitions and validated scoring systems in children and adults (52). A recent consensus initiative proposed uniform approaches in key areas, including where, when, and how many biopsies should be taken; their preparation and processing; histologic scoring; and agreed definitions of response and remission (53). Classic histologic signs may be less commonly identified in colonic biopsies of children compared to adults, especially in those children under the age of 10 years (4, 13, 40). Scoring tools ideally should discriminate between quiescent disease activity and histologic normalization, but not all indices include such necessary detail. Following a Cochrane analysis, the indices with most robust validation date in adult settings are the Nancy Index (NI), Robards Histopathological Index (RI), and Geboes score (GS), although the latter has not undergone formal responsiveness testing (54–57). Their characteristics are summarized in Table 2. The recent ECCO position paper on histopathology in ulcerative colitis recommended use of either the RI or NI in clinical studies (58). Kovach et al. showed fair to moderate inter-observer reproducibility of GS parameters and a positive relationship between endoscopy and histologic features in a pediatric setting, although this was not linear (59). The PROTECT study used a non-validated 5-point scale for categorizing grades of acute and chronic inflammation for a pediatric cohort and found a non-linear association between the histological score and MES in that study (31). With further validation in pediatric settings, the GS seems a better placed tool for use in pediatric research, if not clinical care also.

### Baseline Histology Predictors

There are even less histopathologic data than endoscopic data on predictors of future clinical outcomes in pediatrics. Histology did not feature significantly in the recent PIBD Ahead review of prognostic indicators, due to limited data availability (19). In adults, a higher baseline GS is significantly associated with risk of later clinical relapse (60). Mucosal eosinophilic infiltration and peripheral eosinophilia were associated with clinical severity and corticosteroid therapy use but not with long-term risk for step-up therapy or colectomy in a retrospective pediatric study (61). Conversely in the PROTECT cohort, using a study specific scoring index with GS features, low rectal eosinophil counts(<32/hpf) and villiform surface mucosa at diagnosis were associated with treatment escalation by week 12, and anti-TNFα escalation by week 52 (20, 32). Patients with high rectal eosinophil counts were more likely to have SSFR at 52 weeks. There was no correlation between the endoscopic and histologic severity (31). Unlike the majority of adult studies using multiple biopsies, the PROTECT study looked at a single rectal biopsy from pediatric patients at the time of diagnosis. Histological features associated with poorer clinical outcomes in adults (shorter time to relapse, relapse risk, colectomy, severe UC) include crypt atrophy, basal plasmacytosis, crypt abscess formation and lymphoid follicles (11, 25, 62). Architectural features are not included in the NI or RI, and potentially meaningful predictors may be lost if scores alone are the sole focus of histological predictors of clinical outcomes.

### Histologic Healing and Normalization

Two recent meta-analyses concluded that histologic activity is an impactful variable in predicting clinical outcomes (34, 35). The risk of relapse was higher in patients with histologically active but endoscopically normal appearing disease (OR 2.41; 95% CI, 1.91–3.04) (34). Notable limitations here included significant study heterogeneity, not all studies used validated histopathological score systems, studies with longer term outcomes had greater effect size, and inconsistent predefined scoring system cut-offs were used, even with validated scales. Yoon et al. calculated the annual risk of clinical relapse in patients achieving dual endoscopic and histological normalization at 5% (95% CI, 3.3–7.7%) (35). Clinical outcomes did improve sequentially, depending on whether patients' highest level of attainment was clinical, endoscopic, or histologic remission. Persistent histological disease activity, scored using the NI in a retrospective cohort, was recently associated with a higher 5- year risk of surgery and hospitalization than those in histological remission (37). Although clinically relevant observations, their underpinning milestones cannot yet be assumed to be valid targets for treatment.

The STRIDE-II guidance from the International Organization for the study of IBD proposed histologic normalization as an adjunct to endoscopic healing (MES = 0, UCEIS <1), rather than an independent treatment target itself (63). Without data from prospective treat-to-target trials, it is difficult to justify empiric escalation of medical treatments simply to achieve histological healing. Histologic remission certainly reflects a deeper level of healing in UC, and may still influence therapeutic decisions when identified.

### Primary Sclerosing Cholangitis-Ulcerative Colitis

Patients with primary sclerosing cholangitis-ulcerative colitis (PSC-UC) warrant special mentioning in the context of risk prediction. Although often clinically milder at diagnosis, and less prone to clinical exacerbation, those with PSC-UC are more likely to have persistent subclinical endoscopic and histologic activity, especially in the proximal colon (64). Segmental UCEIS scores, but not MES scores, were significantly higher in patients with PSC-UC compared with UC-alone, particularly for the domains of vascular pattern and ulceration in the largest pediatric study to date (64). As the PUCAI score underestimates persistent endoscopic and histological activity in this patient subgroup, fecal calprotectin is a useful adjunct, with levels <100 μg/g indicative of quiescent histological activity (33). The risk of colectomy in pediatric PSC-UC remains lower than UC-alone and is not predicted by endoscopic disease severity (29). These data provide important clinical messages in PSC-UC: that PUCAI is not a reliable indicator of mucosal remission in this patient subgroup, and that a low threshold for assessing mucosal remission in PSC-UC should be maintained, given their life-long malignancy risks.

## Future Directions

**Figure 1:**
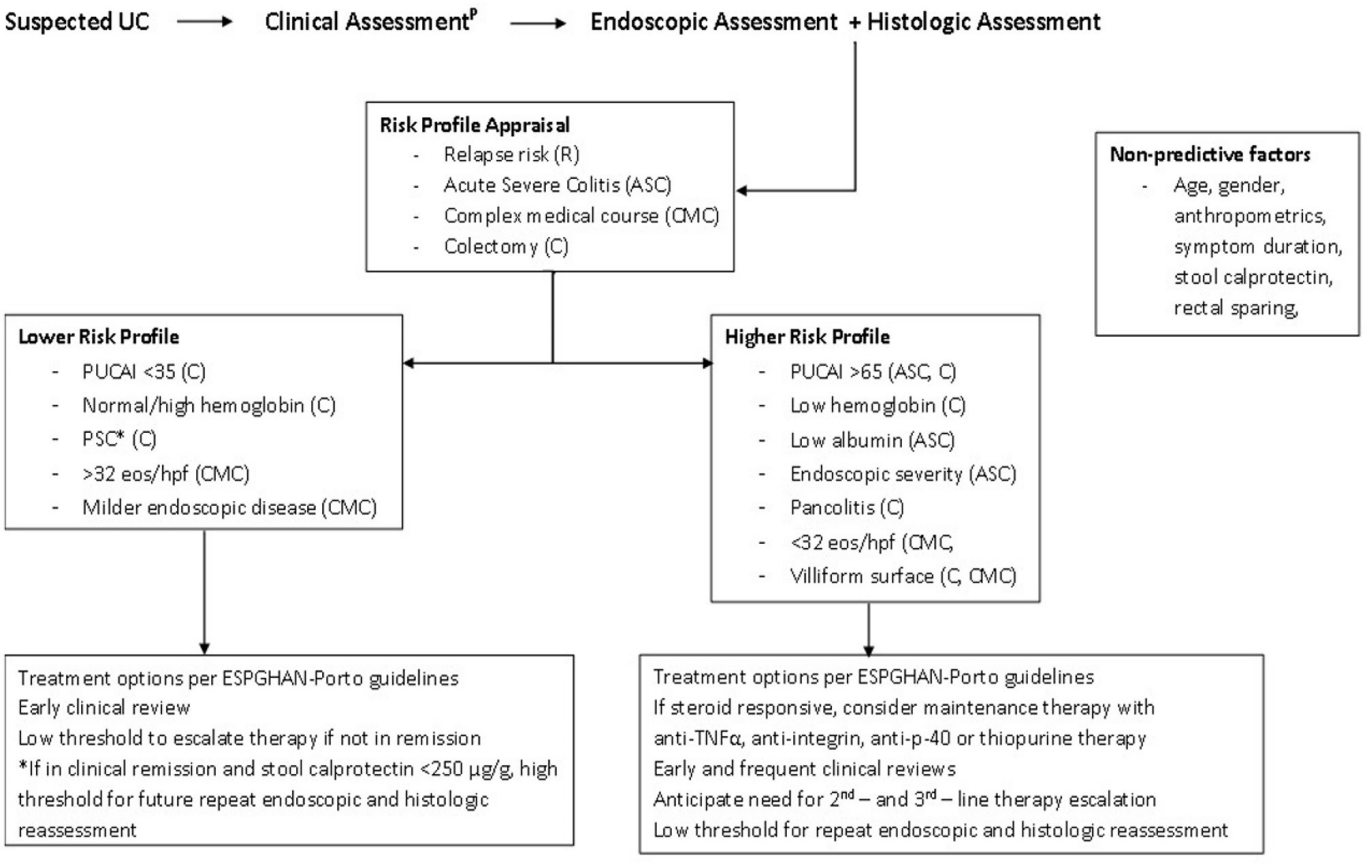
Patient risk profile pathway. *PSC increases the lifetime risk of malignancy. Ongoing endoscopic and histologic disease activity is described in those with PSC-UC, despite reassuring PUCAI scores. Consider more frequent monitoring using calprotectin and/or endoscopy. ^P^According to the IBD Porto guidelines. PUCAI, Pediatric Ulcerative Colitis Activity Index; PSC, Primary Sclerosing Cholangitis; eos/hpf, rectal eosinophils per high powered field; ESPGHAN, European Society for Pediatric Gastroenterology, Hepatology and Nutrition (65, 66).

Opportunities to address the “known unknowns” of risk prediction must be grasped in prospective research initiatives. Pediatric investigator-led research endeavors are often multi-center and collaborative in nature, but retrospective. Clinicians are likely already considering endoscopic and histologic findings in treatment choices—(Figure 1) (65, 66). We recommend incorporating standard reporting approaches in routine clinical care to improve clinical and research quality and, on balance, the UCEIS and GS seem best placed to meet pediatric needs.

Validation of traits, timing, and thresholds for response and remission in children is as necessary as the inclusion of pediatric patients in trials itself. Early time point features are unlikely to predict clinical outcomes beyond the initial 2–4 years after diagnosis. Serial assessments may offer more extended outcome forecasts, and pediatric specialists may need to revisit barriers to endoscopic reassessment if substantial benefits outweigh minimal risks. Resolving the discordance between endoscopic and histologic indices, especially in moderate disease settings, is also necessary before predictive extrapolation should be made.

Head-to-head studies are needed to determine if absolute endoscopic healing alone confers comparable outcomes to histologic healing in children, but this may be challenging in reality. Endoscopic remission will not always correlate with histologic remission, and the number needed to treat with a given therapy to achieve such “dual remission” is likely to be prohibitive. Whether specific histologic atypia or a generalized “incomplete healing” are better predictors of outcomes than endoscopic healing alone could be answered in pediatric settings, with appropriate study design.

Future UC trials need to incorporate valid histopathological outcomes in their design. A signal favoring histologic remission as a portend of reduced relapse risk in adults is clear and consistent. The lack of fully validated pediatric histologic indices challenges the pediatric leadership in the field to address this limitation.

Histologic normalization is more a bonus than a realistic target at present. Whereby strong predictors of refractory severe disease are identified, with poor likelihood of response to currently available medical therapies, earlier consideration of surgical rather than sequential futile medical options should also be considered to avoid unnecessary risk exposure. Alternatively, histological and endoscopic healing as outcome predictors, rather than treatment targets, may be better utilized and investigated as tools to either identify children needing less-intensive therapies, or safe de-escalation of long-term therapies.

Advanced imaging modalities such as magnification colonoscopy (MC), narrow band imaging (NBI), autofluorescence imaging (AFI), chromoendoscopy, confocal laser endomicroscopy (CLE), and endocytoscopy (EC) have shown early promise in UC and may help to bridge the gap between endoscopic and histologic healing in time (67, 68). Large-scale clinical studies are needed to ascertain the relevance of these techniques to clinical outcomes. Complimentary ‘artificial intelligence' technology will likely further enhance the interpretive power of current imaging.

## Conclusion

Predicting the course of UC in children following diagnosis is a reasonable but elusive expectation of patients and clinicians. Current pediatric evidence is based largely on studies which were poorly designed and controlled until recently. Clinicians are pivotal to improving our reporting standards and research outputs, while ensuring that novel predictors of good outcomes are not used as targets of treatment without appropriate validation. For now, available scoring systems are not the prognostic tools they seem to be in adult patients, and their validation has shown promise rather than fruition to date. The unmet potential of readily available endoscopic and histological information should encourage gastroenterologists and pathologists to foster even closer collaborative efforts to advance this field.

## Author Contributions

LS was involved in drafting. SH was involved in all stages of the paper from conceptualization to drafting and final editing. SH is the article guarantor. Both authors contributed to the article and approved the submitted version.

## Conflict of Interest

The authors declare that the research was conducted in the absence of any commercial or financial relationships that could be construed as a potential conflict of interest.
